# Analysis on Machining Performance of Nickel-Base Superalloy by Electrochemical Micro-milling with High-Speed Spiral Electrode

**DOI:** 10.3390/mi10070476

**Published:** 2019-07-16

**Authors:** Yong Liu, Xiaodong Xu, Chunsheng Guo, Huanghai Kong

**Affiliations:** 1 Associated Engineering Research Center of Mechanics & Mechatronic Equipment, Shandong University, Weihai 264209, China; 2 Shenzhen Research Institute of Shandong University, Virtual University Park, Nanshan 518057, Shenzhen, China

**Keywords:** electrochemical milling, high-speed rotation, spiral electrode, finite element analysis, micro-graphic structures

## Abstract

As one of the most promising micro-machining methods, electrochemical micro-machining is widely used in the field of metal micro-structures. The electrochemical micro-milling on Nickel-base superalloy by using high-speed spiral electrode was studied in detail. Firstly, the electric field and flow field models of micro-electrochemical milling are established and analyzed by the finite element method. Then, the milling profile was predicted and the effect of high-speed rotation of electrodes on electrolyte promotion and secondary electrolysis prevention were analyzed. Secondly, the influence of the main machining parameters, such as rotating speed, electrical parameters, and feed rate on machining precision and efficiency was analyzed experimentally. Finally, by optimizing the machining parameters, a series of micro-graphic structures with a width of about 150 μm were obtained on Nickel-base superalloy 718 by using the spiral electrode with a diameter of 100 μm. The experimental and simulation results show that the high-speed rotation of electrodes can greatly improve the machining efficiency and stability. It was proved that micro-electrochemical milling with the high-speed rotating electrode technique is an effective method for machining micro-metal parts.

## 1. Introduction

With the development of new and high technology, the demand for metal micro-structures is gradually increasing. Especially with the development of MEMS, various countries continue to increase their research on micro-machining technology due to its application and huge development prospects [1,2,3,4]. As a very promising micro-machining method, micro-electrochemical machining has been favored by researchers from all over the world due to its excellent machining characteristics. As a combination of numerical control technology with micro-electrochemical machining technology, the electrochemical micro-milling (EMM) method can be used to realize the machining of complex micro-structures by using simple electrodes.

In recent years, many scholars have made relevant research on the electrochemical micro-milling method and have achieved some results. For example, Rolf Schuster et al. [5,6] proposed the use of a nanosecond pulse power supply for machining a tongue-like structure with a thickness of 2.5 μm on a Cu plate by using a columnar electrode of 10 μm. Kock et al. [7] improved the machining accuracy to 100 nm by using picosecond pulse power to fabricate a depth of 5 μm micro-spiral structure on a nickel plate. Staemmler et al. [8] proposed a combination of high-speed cutting and EMM with ultrashort voltage pulses. First, conventional high-precision milling is used to manufacture the main structure. Afterwards the structure is finished by the EMM process. Klocke et al. [9] used the method of multi-physics coupling analysis to simulate and analyze the effect of temperature on machining precision and machining efficiency in the micro-electrochemical machining process. Kim et al. [10,11] reduced the taper of side wall by using nanosecond pulse power and disk-electrode in the EMM process and got a three-dimensional (3D) hemispherical micro-structure with diameter of 60 μm successfully, which showed that the EMM method can achieve higher shape accuracy. Liu [12,13] applied the stratification technology to the EMM process and established the EMM process model, a stepped cavity with a depth of 45 μm and a single-step width of 15 μm was successfully machined. Yuan [14] used current feedback to electrode positioning, and successfully fabricated array grooves with the width of 6 μm. Xu [15] added an adjustable inductance element in the equivalent circuit of the electrochemical micro-machining to form a coupled fluid-electric circuit. With the tungsten electrode of the diameter of 15 μm, the multi-order 3D micro-structure was obtained. The micro-groove width was 16.8 μm, and the machining precision reached 900 nm. Kozak et al. [16] mathematically modeled the process of high-frequency pulse EMM and numerically simulated the contour changes of the anode surface during the EMM process. Rathod et al. [17] conducted experiments of EMM with the disk electrode and fabricated a series of micro-grooves with inverted conical, barrel, spherical, stepped, and inner pocket cross-sections successfully. Liu et al. [18] carried out sets of experiments on Nickel-based super alloys by EMM, and the effects of key parameters on machining localization and surface roughness were analyzed in detail. By using the optimized machining parameters, some 2D complex shapes and 3D square cavity structure with good shape precision and good surface quality were successfully obtained.

Although the above research has its own achievements in the theory or experiment of EMM, there is still a lack of simulation analysis of flow field or electric field in the EMM process. At present, the EMM technology has a long distance from industrial application due to its low machining efficiency. The innovation of this paper is that the electric field and flow field models of micro-electrochemical milling are established and analyzed by the finite element method, which lays a foundation for the follow-up experiments. Meanwhile, by using high-speed spiral electrode, the efficiency and stability of EMM are greatly improved, which makes it more industrialized.

The paper studied EMM with high-speed rotating spiral electrode theoretically and experimentally. Firstly, the electric field and flow field models of EMM are established and analyzed by using the finite element method. Then, the milling profile is predicted and the effect of high-speed rotation of electrodes on electrolyte promotion and secondary electrolysis prevention were analyzed. Secondly, the influence of rotating speed on maximum feed rate is analyzed experimentally, which verifies the effect of high-speed rotation of electrodes on electrolyte renewal and machining efficiency. The influence of the main machining parameters on the groove width was also discussed experimentally. Finally, some micro-graphic structures of the Nickel-base superalloy 718 was machined by using the optimized parameters.

## 2. Principle of Electrochemical Micro-Milling

**Figure 1:**
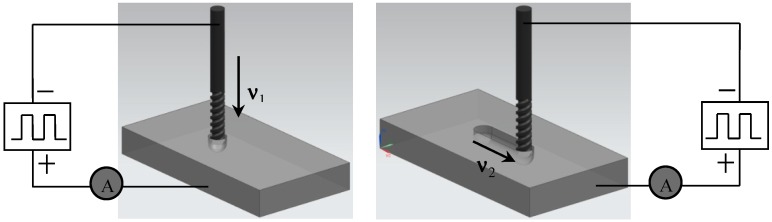
Process of micro-electrochemical milling.

The process of EMM includes longitudinal electrochemical drilling and horizontal electrochemical milling. In the machining process, the depth of machining is determined by electrochemical drilling and the shape of the micro-structure is determined by horizontal milling. Before machining, the micro-spiral electrode is installed firstly on the spindle chuck, then the workpiece is installed in the electrolyte tank and the electrolyte is added to the tank. When the positions of the electrodes and the workpiece are adjusted, the electrolyte circulation system and spindle air cooling system is started. After turning on the high-frequency pulse power, tool setting parameters are set and then the operation of tool setting is performed. After finishing the tool setting, a certain machining gap is set. During machining, after setting up drilling parameters, a certain depth is fed down with a certain feed rate v_1_. Then after setting up milling parameters, the horizontal milling is carried out with speed v_2_. Figure 1 shows the EMM process.

When the micro-electrochemical milling is at an equilibrium state, the machining side gap $b_{x}$ in X direction can be expressed as [18]:

(1) $$b_{x}=\frac{\omega \kappa (\Phi -\delta E)}{v_{2}}$$

where, κ is the electrical conductivity of the electrolyte, Φ is the supply voltage, δE is sum potential of cathode and anode, and $v_{2}$ is the feed rate of the electrode.

**Figure 2:**
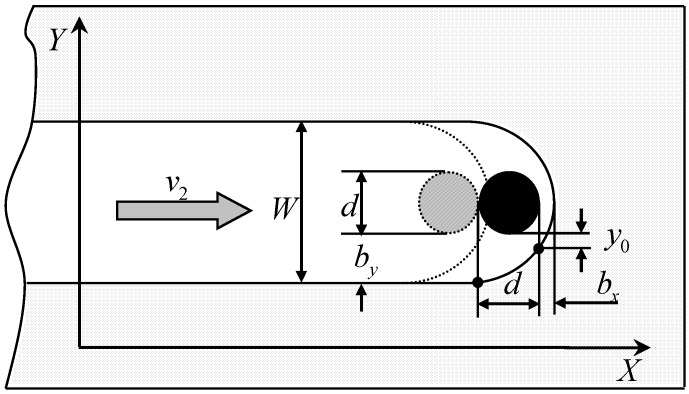
Micro-electrochemical milling in the XY plane.

**Figure 3:**
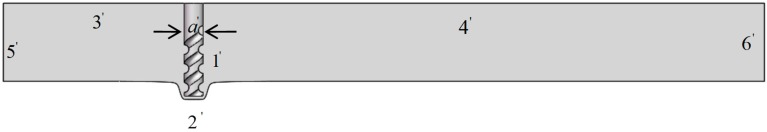
Model of electric field in machining gap.

Figure 2 shows the schematic of micro-electrochemical milling in the XY plane. As shown in Figure 3, the relationship between the side gap and the Y axis can be expressed as:

(2) $$\frac{dy}{dt}=\frac{\omega \kappa (\Phi -\delta E)}{y}$$

Equation (2) can be integrated as follows:

(3) ∫ydy=∫ωκ(Φ−δE)dt

At the initial time, t=0 and $y=y_{0}$. Therefore:

(4) $$\frac{y^{2}}{2}=\kappa \sigma (\Phi -\delta E)t+\frac{{y_{0}}^{2}}{2}$$

When the diameter of the tool electrode is micro-scale, the initial side gap $y_{0}$ can be assumed as $b_{x}$. The machining side gap $b_{y}$ in the Y direction can be obtained as follows:

(5) $$b_{y}=\sqrt{2\omega \kappa (\Phi -\delta E)t+{b_{x}}^{2}}=\sqrt{2\omega \kappa (\Phi -\delta E)\frac{d}{v_{2}}+{b_{x}}^{2}}$$

where, d is the diameter of the tool electrode. The machining gap $b_{x}$ is given in Equation (1).

Therefore, the width of the micro-groove W can be expressed as:

(6) $$W=d+2b_{y}=d+2\sqrt{2\omega \kappa (\Phi -\delta E)\frac{d}{v_{2}}+{b_{x}}^{2}}$$

As shown in Equation (6), the width of the micro-groove depends on numerous parameters, such as the diameter of the tool electrode, feed rate of the tool cathode, material attributes of the workpiece and the tool cathode, parameters of the pulse power supply, and electrical conductivity of the electrolyte.

## 3. Analysis of Electric Field and Flow Field in Electrochemical Micro-Milling (EMM)

### 3.1. Analysis of Electric Field and Current Density

In the EMM process, the area without current density distribution has no substantial influence on the electrochemical machining. Therefore, this paper chooses the electrolyte area with current distribution to analysis. Figure 3 shows the simulation model of the electric field which is in the gap of electrochemical milling. At this point the groove has been machined by electrochemical drilling and the diameter of the micro-mill is a = 0.1 mm. The boundary of the cathode surface is 1, the boundary of the anode surface is 2, and the boundary of the electrolyte is 3, 4, 5, 6.

It is generally considered that the machining process has entered a state of equilibrium when studying the forming process of the electrolytic machining. In this case, the electric field parameter is a function of position and does not change with time. Therefore, the electric field in the machining gap can be regarded as a passive steady current field. And the electrolyte is assumed to be isotropic because the electric field distribution is the only factor to be considered. So, the distribution of potential conforms to the Laplace equation according to the theory of electric field, which is shown as follows:

(7) $$\nabla^{2}\varphi =0$$

In electrochemical machining, both the tool cathode and the workpiece anode are metal conductors, and their potential distribution can be regarded as the equal potential values of different potentials, that is:

(8) φ|Γ1=0

(9) φ|Γ2=Φ

Other boundaries meet the second type of boundary conditions, that is:

(10) $$\frac{\partial \varphi }{\partial n}|\Gamma 3,4,5,6=0$$

COMSOL Multiphysics software was used to simulate. Firstly, the finite element model was established, and the material properties were defined. Then the cathode was set as the electrode boundary and the potential of cathode was 0 V, which satisfied the equation:

(11) $$\phi_{s}-\phi_{a}=E_{eq}$$

where, $\phi_{s}$is the cathode potential, and $E_{eq}$is the equilibrium potential between the metal electrode and the electrolyte, which is measured on the electrode surface at a common reference potential. The anode is set as the boundary condition of the external plating electrode, which is connected to the nanosecond pulse power supply. The frequency of the nanosecond pulse power is 2 × 10^5^ Hz and the duty cycle is 9%. So, the pulse period is 5 μs, the pulse width is 450 ns, the voltage amplitude is set to 5.5 V, and electrolyte conductivity is 11.6 S/m.

In addition to the surface of the anode and the cathode, the other boundaries are set as insulation boundary conditions, that is:

(12) $$\vec{i}\cdot \vec{n}=0$$

The initial gap between the cathode and the surface of the machined workpiece is 5 μm, the type of user-controlled grid is selected to mesh the model, and a free mesh of triangles is added. To eliminate mesh size influence, the mesh quantity increased gradually until the simulation results no longer change.

**Figure 4:**
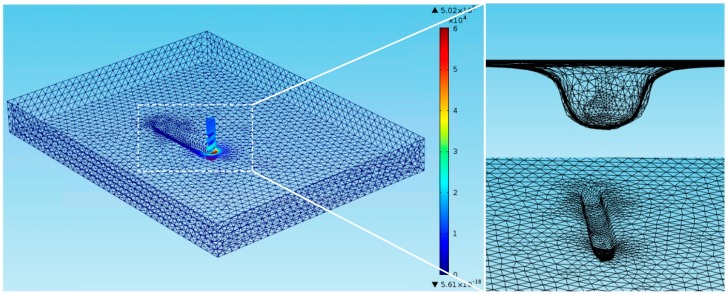
Distribution of electric field intensity in the machining gap when machining time is 30 min.

As shown in Figure 4, during electrochemical machining, the distribution of electric field intensity in the machining gap is uneven. The strength of the electric field is high at the two corners and the lowest point of the groove, and the strength around them is low. That is mainly caused by the concentration of ions from anode to cathode. The distribution of the uneven electric field strength makes the etching rate at workpiece surface uneven. The corrosion rate in the concentrated area of the electric field is faster, and the corrosion rate in other areas is slower. Therefore, there will be an isolated island in the process of machining, and the isolated island means that the corrosion rate is faster in the surrounding area and is slower in the middle part. The emergence of the isolated island makes the machining gap decrease, the current density increase, and the amount of metal corrosion increase. Finally, the amount of corrosion on the island will surpass the surrounding parts.

**Figure 5:**
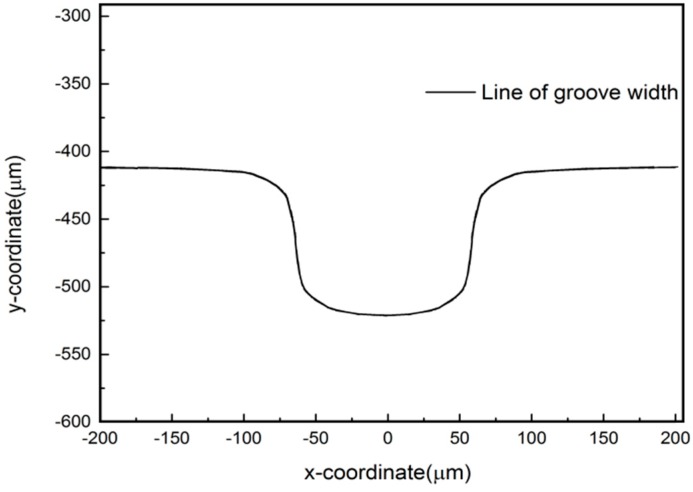
The profile of the groove width when the machining time is 30 min.

From the analysis of Figure 4, it can be seen that the electric field intensity at the machining area is larger, and the electric field intensity in other areas is smaller or even does not exist. Therefore, in the process of continuous feeding of the tool electrode, the surface material of the workpiece will form a groove due to the corrosion and dissolution. In order to predict the groove width during machining, the profile of the groove width is extracted when the machining time is 30 min. As shown in Figure 5, the groove width is about 150 μm.

### 3.2. Analysis of Flow Field in Machining Gap

In order to study the influence of micro-spiral electrodes with high-speed on the accuracy and efficiency of micro-electrochemical milling, the flow field in the machining gap needs to be simulated.

This paper uses CFX software for numerical simulation. The electrolyte is assumed to be an invariable incompressible Newton fluid, and the air satisfies the ideal gas equation of state. The air follows the mass conservation equation, and the electrolyte follows the momentum conservation equation, and the sum of the volume fraction of the two phases is 1.

The turbulent energy transport equation in the direction of X is as follows [19]:

(13) $$\frac{\partial \left(\rho k\right)}{\partial t}+\frac{\partial \left(\rho ku_{i}\right)}{\partial x_{i}}=\frac{\partial }{\partial x_{j}}\left[\left(\mu +\frac{\mu_{t}}{\sigma_{k}}\right)\frac{\partial k}{\partial x_{j}}\right]+\mu_{t}\left(\frac{\partial u_{i}}{\partial x_{j}}+\frac{\partial u_{j}}{\partial x_{i}}\right)\frac{\partial u_{i}}{\partial x_{j}}-\rho C_{D}\frac{k^{\frac{3}{2}}}{\varepsilon }$$

where, *k* is the turbulent energy, ρ is the electrolyte density, $x_{i}$, $x_{j}$ is the form of a tensor symbol in the x direction, u is the velocity, $u_{i}$ and $u_{j}$ are expressed as the form of the tensor symbol, t is a moment when the fluid particle moves, μ is the dynamic viscosity, ɛ is the turbulent energy dissipation rate, $\mu_{t}$ is the turbulent viscosity, $\sigma_{k}$ is the Prandtl constant corresponding to the turbulent energy *k*, and $C_{D}$ is the empirical constant.

The turbulence model selected in this paper is the Standard *k*-*ε* model, and the transport equation corresponding to the *k*-*ε* model [19,20] is:

(14) $$\frac{\partial \left(\rho k\right)}{\partial t}+\frac{\partial \left(\rho ku_{i}\right)}{\partial x_{i}}=\frac{\partial }{\partial x_{j}}\left[\left(\mu +\frac{\mu_{t}}{\sigma_{k}}\right)\frac{\partial k}{\partial x_{j}}\right]+G_{k}+G_{b}-\rho \varepsilon -Y_{M}$$

(15) $$\frac{\partial \left(\rho \varepsilon \right)}{\partial t}+\frac{\partial \left(\rho \varepsilon u_{i}\right)}{\partial x_{i}}=\frac{\partial }{\partial x_{j}}\left[\left(\mu +\frac{\mu_{t}}{\sigma_{\varepsilon }}\right)\frac{\partial \varepsilon }{\partial x_{j}}\right]+C_{1\varepsilon }\frac{\varepsilon }{k}\left(G_{k}+C_{3\varepsilon }G_{b}\right)-C_{2\varepsilon }\rho \frac{\varepsilon^{2}}{k}$$

where, $G_{k}$ is the k-generation of turbulent kinetic energy caused by the average speed gradient, $G_{b}$ is the b-generation of turbulent kinetic energy caused by buoyancy, $Y_{M}$ is the influence of total dissipation rate caused by compressible turbulent pulsatile expansion, $\sigma_{\varepsilon }$ is the Prandtl constant corresponding to the turbulent energy dissipation rate, $C_{1\varepsilon }$, $C_{2\varepsilon }$ are empirical constants, and their values are 1.44 and 1.92 respectively, and $C_{3\varepsilon }$ is the influence of turbulent energy dissipation rate caused by buoyancy.

**Figure 6:**
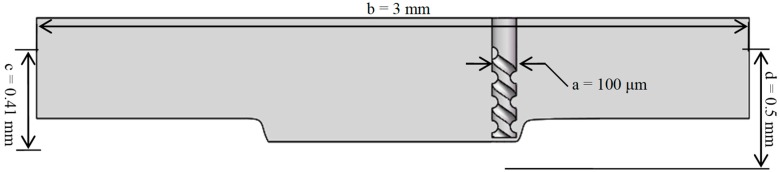
Flow field model of the machining gap.

In this paper, the fluid region is modeled when the machining time is 17 min. The length of the fluid region is b = 4 mm, the depth of the fluid area is c = 0.41 mm, and the length of the micro-spiral electrode is d = 0.5 mm. The flow field model of the machining gap is shown in Figure 6.

When CFX is used to simulate, the solution model is set first, then the material properties are defined, and the boundary conditions are set. The distance between the upper surface of the machining part and the electrolyte level is assumed to be 0.1 mm, the rotation speed of the micro-spiral electrode is set to be 20,000 r/min, and the other wall is set to be the non-slip boundary condition.

In order to analyze the changes of the gap flow field during the process of electrochemical machining, a two-dimensional cross-section of the three-dimensional model is analyzed.

**Figure 7:**
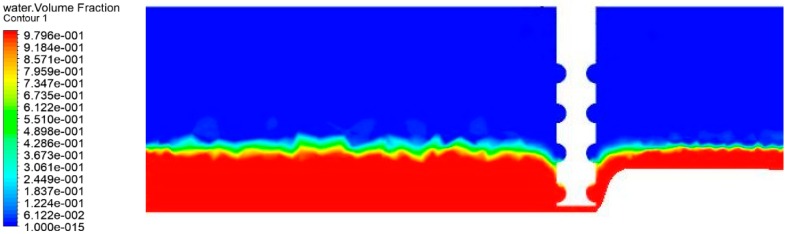
Gas distribution in the micro-machining gap.

Figure 7 shows the two-phase flow of gas distribution in the micro-machining gap, and the micro-machining is driven by a micro-spiral electrode with a diameter of 100 μm at high speed.

From Figure 7, when the spiral electrode is rotating at a high speed, the groove of the spiral electrode will rotate the flow around the electrode, and the axisymmetric vertical vortex flow is formed quickly. Within a certain range, the gas film shown in the above figure is generated due to the increase of gas content around the bottom of the spiral electrode. Gas–liquid distribution in the micro-machining gap by use of the helix micro-electrode with different rotating speeds is shown in Figure S1. The gas film wraps the end of the spiral electrode to prevent secondary electrochemical machining, which plays a good insulation role.

**Figure 8:**
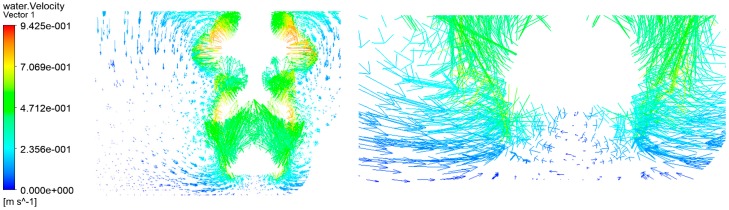
Velocity vector of the flow field in the micro-machining gap.

Figure 8 shows the velocity vector of the flow field in the micro-machining gap, and the micro-machining is driven by a micro-spiral electrode with a diameter of 100 μm at the speed of 20,000 r/min.

From Figure 8, the direction of the fluid velocity is oblique at the side of the micro-spiral electrode, and the direction of the velocity is downward along the wall of the micro-spiral electrode. The vertical axis vortex flow not only has tangential and radial velocity, but also has an upward axial velocity. The spiral flow is formed due to the existence of the axial pressure gradient and the inner cylinder rotation, and the spiral flow is similar to the vertical swirling vortex, which is the synthetic motion of circumferential vortex and axial annulus flow. It can bring out the electrolytic products due to its axial velocity. Since the vortex tail has a negative pressure, the electrolysis product in the machining gap can be carried out spirally along the axial direction with electrolytes. At the same time, the negative pressure can also absorb the fresh electrolyte outside the machining area and can make it enter into the machining gap along the side wall. Therefore, the electrolysis products can be discharged quickly and the electrolyte can be updated rapidly.

## 4. Results and Discussion

Sets of experiments were carried out by using the EMM experimental system. The system uses a micro-spiral cylindrical WC (tungsten carbide) electrode with a diameter of 100 μm as a cathode and uses a Nickel-base superalloy 718 with a thickness of 500 μm as an anode. The NaNO_3_ solution with a mass fraction of 5% was used as an electrolyte and a high-frequency pulse power source with a maximum frequency of 50 MHz was used to provide energy. Experiments of the micro-electrochemical milling of a Nickel-base superalloy 718 plate were carried out to demonstrate the effects of machining parameters on the groove width. As mentioned above, the groove width *W* is considered as the evaluation of machining localization. The groove width is dependent on the pulse voltage, pulse width, feed rate, and so on. Table 1 shows the machining conditions for EMM experiments.

### 4.1. Influence of Rotating Speed on Maximum Feed Rate

**Figure 9:**
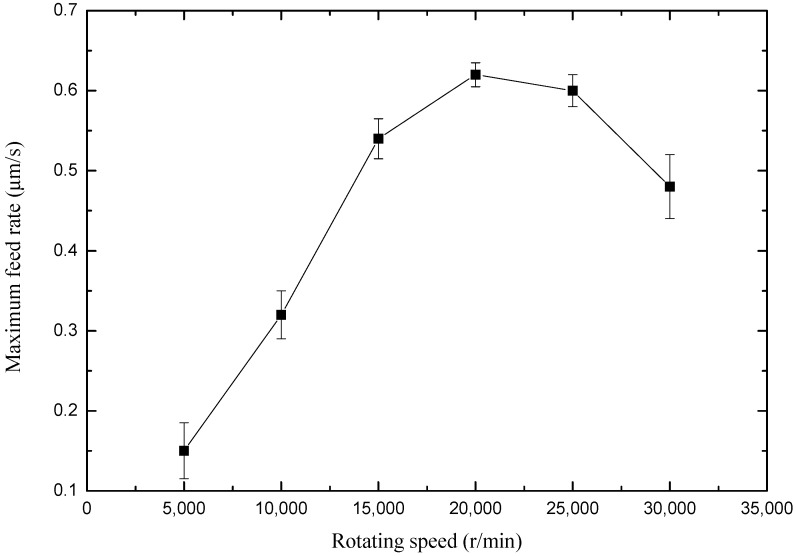
Maximum feed rate along with rotating speed of the micro-spiral electrode.

In order to study the influence of micro-spiral electrode rotating speed on maximum feed rate, a set of comparative experiments were carried out. The experimental parameters were as follows: peak voltage of 5.5 V, pulse period of 5 μs, and pulse on time of 450 ns. Figure 9 shows the variation of maximum feed rate with rotating speed varying from 5000 r/min to 30,000 r/min. Because the electrolyte can be updated rapidly and electrolysis products can be removed in time with the high rotating speed of the micro-spiral electrode, the electrochemical reaction is accelerated.

It can be found that the maximum feed rate allowed for the tool electrode can be obviously improved with rotating speed from 5000 r/min to 20,000 r/min. Because the machining localization is improved with the feed rate of tool electrode, the high-speed micro-spiral electrode also has an advantage to the machining localization. On the other hand, the high-speed spiral electrode forms a zone of negative pressure near the electrode, and air is gathered around the electrode gradually. When the rotating speed of the micro-spiral electrode is faster than 25,000 r/min, there is not enough electrolyte around the electrode which would limit the speed of electrochemical milling. Therefore, only an appropriate rotating speed of the micro-spiral electrode can make micro-electrochemical milling more efficient. The suggested rotating speed of the micro-spiral electrode is 20,000 r/min.

### 4.2. Influence of Peak Voltage on Machining Precision

**Figure 10:**
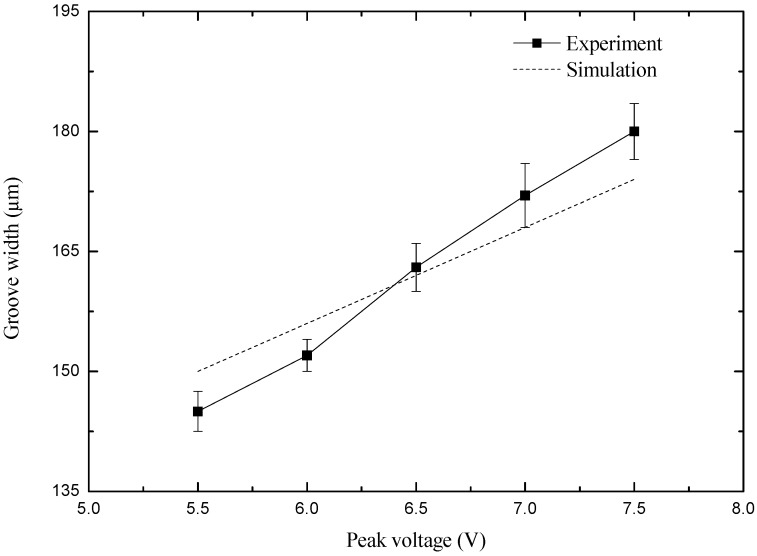
Curve of groove width with the peak voltage.

In order to study the effect of voltage amplitude on the precision of electrochemical milling, a group of comparative experiments have been carried out. The experiment parameters are as follows: the pulse period is 5 μs, the pulse width is 450 ns, the electrode speed is 20,000 r/min, the diameter of the micro-spiral electrode is 100 μm, the drilling depth is 100 μm, and the milling speed is 0.5 μm/s. The curve of groove width with peak voltage in simulation and experiment results is shown in Figure 10, when the peak voltage is varying from 5.5 V to 7.5 V.

As shown in Figure 10, the groove width increases as the peak voltage increases from 5.5 V to 7.5 V when other parameters are unchanged, and the above parameters are approximately linear. The results of the machining at different peak voltage are shown in Figure 14a by scanning electron microscope (SEM). Figure 14a shows that when the peak voltage is 5.5 V, the machined micro-groove has a smaller width of 135 μm and has higher dimensional precision. When the peak voltage is 7.0 V and 7.5 V, the machined micro-groove is wider, and the groove width is 170 μm at 7.5 V. At the same time, there is a certain amount of stray corrosion, which is in place at the beginning of machining.

Therefore, a high-quality micro-groove can be obtained with a smaller peak voltage. However, it is difficult to work when the voltage is too small, because the electrolytic etching ability is insufficient when the voltage is too small, and it is easy to short-circuit when the feed rate is constant. So, under the current parameters, the more suitable voltage is 5.5 V for stable machining.

### 4.3. Influence of Pulse Width on Machining Precision

**Figure 11:**
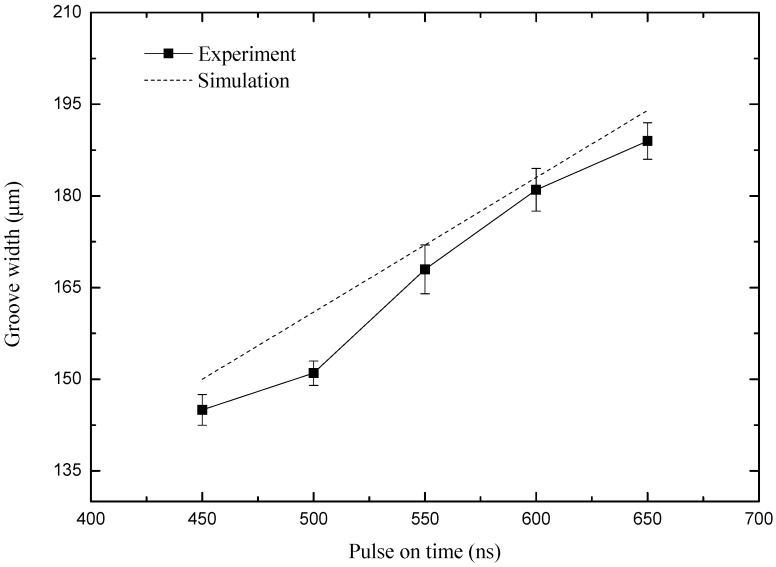
Curve of groove width with the pulse width.

In order to study the effect of pulse width on the precision of electrochemical milling, a group of comparative experiments were carried out. The experimental parameters were as follows: the pulse width is varying from 450 to 650 ns, and the other parameters are the same as the above experiments. The curve of the groove width with pulse width in simulation and experiment results is shown in Figure 11.

As shown in Figure 11, the groove width increases as the pulse width increases from 450 ns to 650 ns when other parameters are unchanged, and the above parameters are approximately linear. The results of the machining at different pulse width are shown in Figure 14b by SEM. It can be seen from Figure 14b when the pulse width is 450 ns, the machined micro-groove has a smaller width of 135 μm and has higher dimensional precision. When the pulse width is 500 ns, the machined micro-groove has a slightly wider width of 141 μm and has a better overall consistency. When the pulse width is 650 ns, the machined micro-groove has a very wide width of 179 μm.

Therefore, a high-quality micro-groove can be obtained with a smaller pulse width. However, it is difficult to work when the pulse width is too small. Because the electrolytic etching ability will be reduced when the pulse width is too small, and it is easy to short-circuit when the rate of erosion is smaller than the feed rate. So, under the current parameters, the more suitable pulse width is 450 ns for a smaller micro-groove.

### 4.4. Influence of Pulse Period on Machining Precision

**Figure 12:**
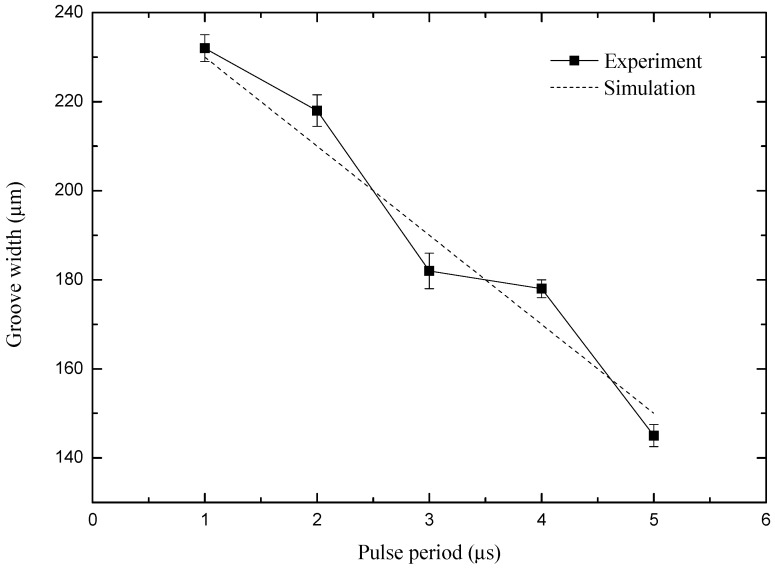
Curve of groove width with the pulse period.

In order to study the influence of pulse period on machining precision, a set of comparative experiments were carried out. The test parameters were as follows: peak voltage of 5.5 V, pulse on time of 450 ns, feed rate of the micro-spiral electrode is 0.5 μm/s, and rotating speed of the micro-spiral electrode is 20,000 r/min. Figure 12 shows the variation of micro-groove width with pulse period varied from 1 μs to 5 μs in simulation and experiment results.

As shown in Figure 12, the width of the micro-groove decreases linearly with the increase of the pulse period. The experiment results show that the larger pulse period can obviously reduce the stray corrosion, meaning that the machining localization becomes well as the pulse period increases. Therefore, the pulse period should be as large as possible under the condition of a steady machining process. Under the conditions of the test parameters above, the most appropriate pulse period is 5 μs. As is shown in Figure 14c, when the pulse period is 5 μs and the other parameters are unchanged, the quality and machining stability of the machined groove are better.

### 4.5. Influence of Feed Rate on Machining Precision

**Figure 13:**
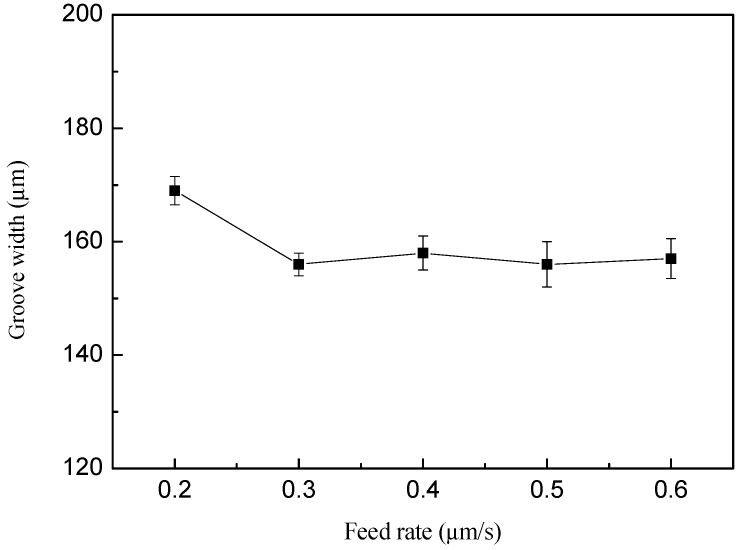
Curve of groove width with the feed rate.

In order to study the effect of feed rate on the precision of electrochemical milling, a group of comparative experiments were carried out. The experimental parameters were as follows: the feed rate is varying from 0.2 to 0.6 μm/s, and the other parameters are the same as the above test. The curve of the groove width is shown in Figure 13.

As shown in Figure 13, the groove width increases as the feed rate increases from 0.2 μm/s to 0.3 μm/s when the other parameters are unchanged. The groove width does not change basically when the speed is greater than 0.3 μm/s. The possible reason for this phenomenon is that at a relatively slow feed rate, the secondary electrolysis process takes a longer time and the groove width is larger. However, when the feed rate is greater than a certain value, the groove width reaches the minimum value of the electrical machining parameters and basically remains unchanged.

**Figure 14:**
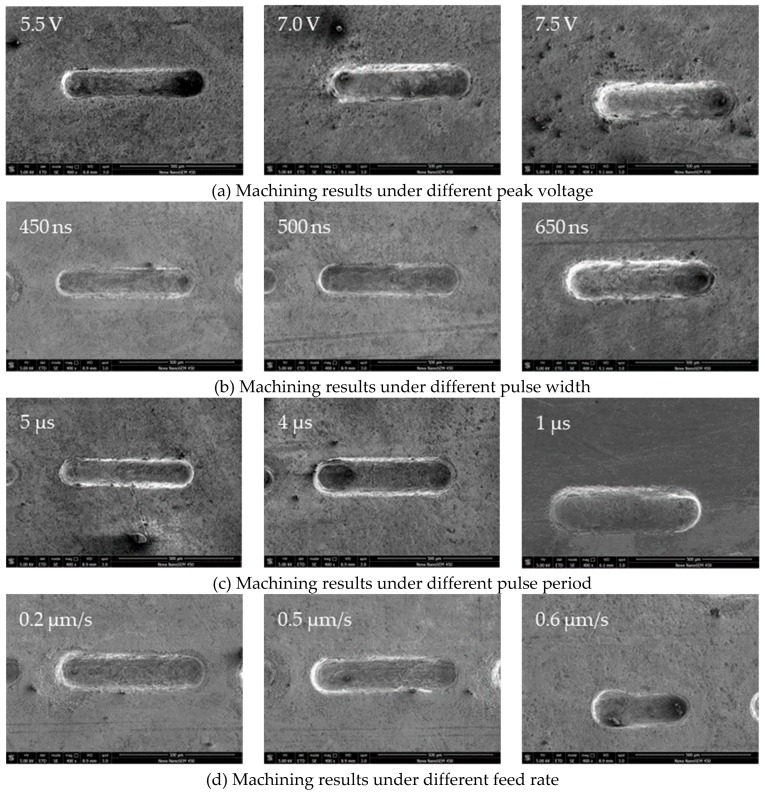
Machining results under different parameters.

The results at different feed rate are shown in Figure 14d by SEM. As is shown in Figure 14d, when the feed rate is 0.2 μm/s, the width of the groove is wider, which is 169 μm. At the same time, there will be stray corrosion around the groove. When the feed rate is 0.6 μm/s, the groove width is smaller. However, it is easy to short-circuit after a period of machining. So, the more suitable feed rate is 0.5 μm/s for the machining micro-groove.

### 4.6. Machining Results

**Figure 15:**
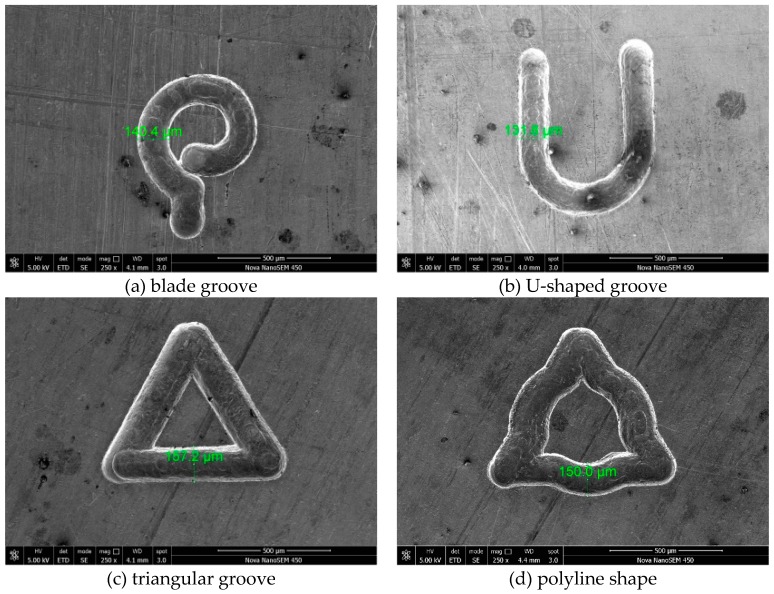
Typical machining results.

A series of micro-graphic structures were obtained by optimizing the parameters. The SEM pictures of micro-graphic structures are shown in Figure 15. The groove width of the machined pattern is about 150 ± 10 μm. The machining parameters of the above figures are as follows: the machining voltage is 5.5 V, the pulse period is 5 μs, the pulse width is 450 ns, the horizontal milling feed rate is 0.5 μm/s, and the electrode rotation speed is 20,000 r/min.

It can be seen from the machining results that the EMM technique can machine any straight line, arbitrary curve, and the combination of straight line and curve on metals, which shows the powerful capability of machining complex structures after optimizing the machining parameters.

## 5. Conclusions

The paper studied electrochemical micro-milling with high-speed rotating spiral electrode through the simulation and experimental methods. The conclusions are as follow:

(1) The electrochemical micro-milling model with high-speed rotating was established based on the finite element analysis method, and the change of workpiece surface profile is predicted by the simulation of machining electric field. Through the simulation of machining flow field, it was found that the gas core formed around the electrodes has a role of electrolyte promotion and secondary electrolysis prevention.
(2) The influence of the key machining parameters on the machining precision was studied through experiments. The experimental results show that EMM localization can be improved by using the power supply of low peak voltage, high frequency, and short pulse on time.
(3) By optimizing the machining parameters and using the spiral electrode with the diameter of 100 μm, a series of micro-graphic structures with the width about 150 μm were obtained on the Nickel-base superalloy 718, which demonstrates that EMM with high-speed spiral electrode is a highly promising technology for fabricating metal complex micro-structures with higher machining efficiency and stability.
(4) Because of the high-speed rotation of the electrodes, the size of the electrode was limited and can not be too small in the present study. In the near future, the effect of ultrasonic vibration-assisted EMM on the improvement of machining quality could be studied in detail.

## Figures and Tables

**Table 1:** Machining conditions for EMM experiments.

Parameters	Values
Applied voltage (V)	5.5–7.5
Pulse width (ns)	450–650
Pulse period (μs)	1–5
RRotational speed (r/min)	55,000–30,000
Cathodal electrode	100 μm WC electrode
Workpiece material	Super alloy 718
Electrolyte	5% NaNO_3_ solution
Feed rate (μm/s)	0.2–0.6

## Supplementary Materials

The following are available online at https://www.mdpi.com/2072-666X/10/7/476/s1, Figure S1: Gas-liquid distribution in micro machining gap by use of the helix micro electrode with different rotating speeds.
