# Supplementary material for: Analysis on Machining Performance of Nickel-Base Superalloy by Electrochemical Micro-milling with High-Speed Spiral Electrode
Source: Micromachines (Basel). 2019 Jul 16;10(7):476. doi: 10.3390/mi10070476 (PMC6680569; doi:10.3390/mi10070476)
Supplement: Supplementary file 1 [file micromachines-10-00476-s001.pdf]

Supplementary Materials: Analysis on Machining Performance of Nickel-base Superalloy by Electrochemical Micro-milling with High-Speed Spiral Electrode

Yong Liu, Xiaodong Xu, Chunsheng Guo and Huanghai Kong

Figure S1 is the diagrams of gas-liquid distribution in micro machining gap by use of the helix micro electrode with rotating speed from 5000 r/min to 40,000 r/min. With the increase of the electrode rotating speed, more and more air was gathered around the electrode at the air-electrolyte interface and formed a gas core. When the rotating speed of micro helix electrode was greater than 20,000 r/min, the gas core almost covered the whole electrode as shown in Figure S1d and Figure S1e, and the oversize gas core played a role of insulating layer of the electrode which may result in the interruption of ECM. Therefore, it is very important to select an appropriate rotating speed of the micro helix electrode in micro electrochemical milling.

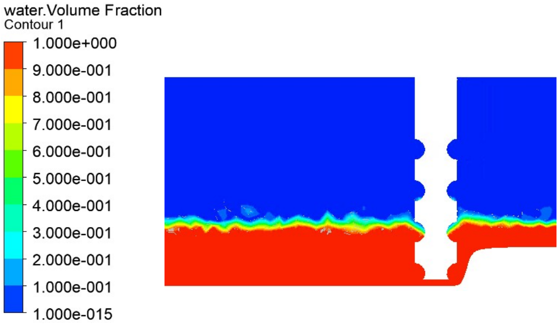

(a) 5000 r/min

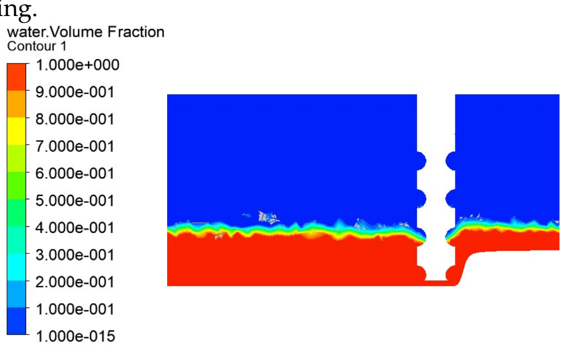

(b) 10,000 r/min

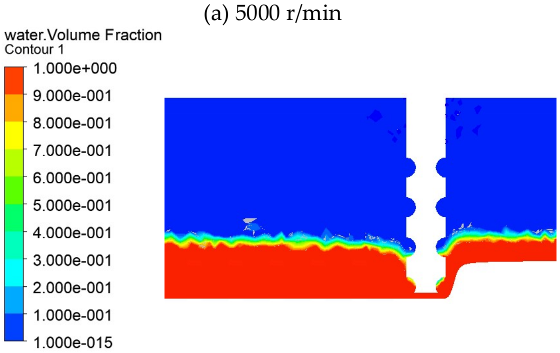

(c) 20,000 r/min

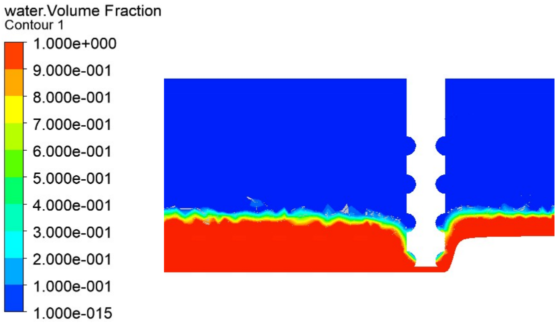

(d) 30,000 r/min

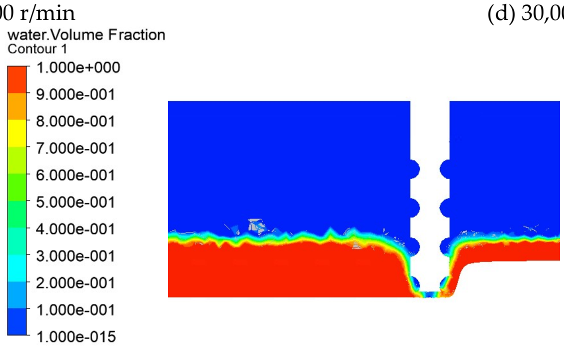

(e) 40,000 r/min

Figure S1. Gas-liquid distribution in micro machining gap by use of the helix micro electrode with different rotating speeds.
